# Cholinergic system in patients with chronic kidney disease: cognitive and renal implications

**DOI:** 10.1093/ndt/gfaf029

**Published:** 2025-02-07

**Authors:** Hong Xu, Maria Eriksdotter, Gaye Hafez, Sumonto Mitra, Annette Bruchfeld, Vesna Pešić, Robert Unwin, Carsten A Wagner, Ziad A Massy, Carmine Zoccali, Marion Pepin, Giovambattista Capasso, Sophie Liabeuf, CONNECT Action (Cognitive Decline in Nephro-Neurology European Cooperative Target), Giovambattista Capasso, Alexandre Andrade, Mustafa Arici, Maie Bachmann, Matthew Bailey, Michelangela Barbieri, Mickaël Bobot, Annette Bruchfeld, Inga Arune-Bumblyte, Daiva Rastenytė, Antonello Calcutta, Giovanna Capolongo, Sol Carriazo, Michele Ceccarelli, Adrian Constantin Covic, Ananya De, Pilar Delgado, Nicole Endlich, Matthias Endres, Fabrizio Esposito, Michele Farisco, Quentin Faucher, Ana Carina Ferreira, Andreja Figurek, Denis Fouque, Casper Franssen, Ivo Fridolin, Sebastian Frische, Liliana Garneata, Loreto Gesualdo, Konstantinos Giannakou, Olivier Godefroy, Aleksandra Golenia, Dimitrios Goumenos, Eugenio Gutiérrez Jiménez, Gaye Hafez, Ewout Hoorn, Pedro Henrique Imenez Silva, Raafiah Izhar, Dearbhla Kelly, Shelli Kesler, Aleksandra Klimkowicz-Mrowiec, Samuel Knauss, Justina Kurganaite, Hélène Levassort, Sophie Liabeuf, Jolanta Malyszko, Laila-Yasmin Mani, Gianvito Martino, Ziad Massy, Christopher Mayer, Armida Mucci, Alma Mutevelic-Turkovic, Rikke Nielsen, Dorothea Nitsch, Alberto Ortiz, Vasileios Panagiotopoulos, Despoina Karasavvidou, Giuseppe Paolisso, Bojana Pejušković, Marion Pepin, Alessandra Perna, Andrea Perrottelli, Vesna Pešić, Pasquale Pezzella, Merita Rroji (Molla), Ivan Rychlík, Giorgos Sakkas, Mariadelina Simeoni, Maria José Soler Romeo, Goce Spasovski, Ana Starčević, Gioacchino Tedeschi, Francesco Trevisani, Robert Unwin, Evgueniy Vazelov, Carsten Alexander Wagner, Franca Wagner, Christoph Wanner, Andrzej Wiecek, Hong Xu, Miriam Zacchia, Lefteris Zacharia, Irene Zecchino, Carmine Zoccali, Francesco Mattace-Raso, Karl-Hans Endlich, Norberto Perico, Giuseppe Remuzzi, Francesco Trepiccione, Mark Okusa, Vincenzo Di Marzo, Peter Blankestijn, Kai-Uwe Eckardt, Maximilian Konig, Ron Gansevoort, Hassan Askari, Brian Hansen, Sunna Snaedal, Elena Cuiban, Edoardo Caporusso, Vincenzina Lo Re, Jonathan Roiser, Kerry Rosenberg, Alvino Bisecco, Laura Denby, Onkar Prakash Kulkarni, Kumar Sharma, Subrata Debnath, Afaf Jaafar, Anna Capasso, Michele Mulholland, Biruh Workeneh, Anna Iervolino, Simon Fraser, Isabelle Frey-Wagner, Annachiara Pastore, Romaldas Mačiulaitis, Antonio De Donato, Ana Farinha

**Affiliations:** Division of Clinical Geriatrics, Department of Neurobiology, Care Sciences and Society, Karolinska Institutet, Stockholm, Sweden; Division of Clinical Geriatrics, Department of Neurobiology, Care Sciences and Society, Karolinska Institutet, Stockholm, Sweden; Department of Pharmacology, Faculty of Pharmacy, Altinbas University, Istanbul, Turkey; Paris-Saclay University, UVSQ, Inserm, Clinical Epidemiology Team, Centre de Recherche en Epidémiologie et Santé des Populations (CESP), Villejuif, France; Division of Clinical Geriatrics, Department of Neurobiology, Care Sciences and Society, Karolinska Institutet, Stockholm, Sweden; Department of Health, Medicine and Caring Sciences, Linköping University, Linköping, Sweden; Department of Renal Medicine, Karolinska University Hospital and CLINTEC Karolinska Institutet, Stockholm, Sweden; Faculty of Pharmacy, University of Belgrade, Belgrade, Serbia; Department of Renal Medicine, Royal Free Hospital, University College London, London, UK; Institute of Physiology, University of Zürich, Zurich, Switzerland; Paris-Saclay University, UVSQ, Inserm, Clinical Epidemiology Team, Centre de Recherche en Epidémiologie et Santé des Populations (CESP), Villejuif, France; Association pour l'Utilisation du Rein Artificiel dans la région parisienne (AURA) and Ambroise Paré University Hospital, APHP, Department of Nephrology Boulogne-Billancourt, Paris, France; Biogem Research Institute, Ariano Irpino, Italy; Associazione Ipertensione Nefrologia Trapianto Renale (IPNET), c/o Nefrologia, Grande Ospedale Metropolitano, Reggio Calabria, Italy; Paris-Saclay University, UVSQ, Inserm, Clinical Epidemiology Team, Centre de Recherche en Epidémiologie et Santé des Populations (CESP), Villejuif, France; Department of Geriatrics, Ambroise Paré University Medical Center, APHP, Boulogne-Billancourt, France; Biogem Research Institute, Ariano Irpino, Italy; Department of Translational Medical Sciences, University of Campania Luigi Vanvitelli, Naples, Italy; Pharmacoepidemiology Unit, Department of Clinical Pharmacology, Amiens University Medical Center, Amiens, France; MP3CV Laboratory, EA7517, Jules Verne University of Picardie, Amiens, France

**Keywords:** cholinergic system, chronic kidney disease, cognitive impairment

## Abstract

Cholinergic synapses are widespread throughout the human central nervous system. Their high density in the thalamus, neocortex, limbic system and striatum suggests that cholinergic transmission plays a vital role in memory, attention, learning and other higher cognitive functions. As a result, the brain's cholinergic system occupies a central position in research on normal cognition and age-related cognitive decline, including dementias such as Alzheimer's disease. In addition to its role in the brain, neuronal cholinergic pathways are essential for the physiological regulation of the body’s organs, including the kidneys, through the parasympathetic branch of the peripheral nervous system. Chronic kidney disease (CKD) is a non-communicable disease with a global prevalence of ≈10%. Cognitive impairment is common among patients with CKD, with reported prevalence rates ranging from 30% to 60%, depending on the definitions and assessment methods used. Given the importance of the cholinergic system in cognitive processes, it may be a key area of focus for evaluating cognitive function in this population. In this current narrative review, we will first examine evidence linking the cholinergic system to cognitive functions and then we will discuss the potential implications of cholinergic function in patients with CKD.

## Graphical Abstract

**Graphical Abstract:**
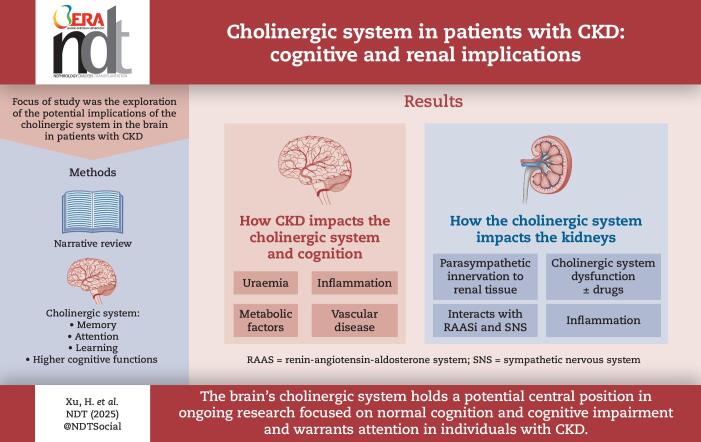

## INTRODUCTION

Chronic kidney disease (CKD) is a progressive condition that affects >10% of the global population, totalling >800 million people [1]. It has become a significant contributor to global mortality and is one of the few non-communicable diseases with an increasing death rate over the past 2 decades [2]. There is mounting evidence that CKD is an independent risk factor for cognitive impairment [3–5]. Even mild cognitive changes can affect essential aspects of healthcare management, including medication adherence, decision-making and active participation in care. This is particularly critical for patients with CKD, since nephrology is often considered one of the most complex medical subspecialties due to the high prevalence of comorbidities and the need to manage polypharmacy [6, 7].

Despite growing evidence, the complex pathogenic relationship and precise regulatory mechanisms linking CKD to cognitive impairment remain unclear and require further investigation. In addition to well-known cardiovascular risk factors such as diabetes, inflammation, hypertension and dyslipidaemia, kidney-specific risk factors, including uraemic toxins, may also increase the vulnerability of CKD patients to neurological disorders [3, 8]. Cholinergic synapses are distributed throughout the human central nervous system (CNS), with high concentrations in the thalamus, neocortex, limbic system and striatum. This distribution indicates that cholinergic transmission plays a crucial role in memory, attention, learning and other higher cognitive functions [3, 9]. Consequently, the brain's cholinergic system remains a focal point of research on normal cognition and age-related cognitive decline, including dementias such as Alzheimer's disease (AD).

The role of the cholinergic system in cognitive impairment among CKD patients warrants closer attention. Emerging evidence also suggests that the cholinergic system may play a role in the pathophysiology of both acute kidney injury (AKI) [10] and CKD [11]. Dysregulation of this system could contribute to the renal dysfunction observed in CKD. Notably, the cholinergic anti-inflammatory pathway, mediated by the vagus nerve, has been shown to exert anti-inflammatory effects throughout the body, including the kidneys [12].

In this narrative review, we will begin with evidence linking the cholinergic system to cognitive functions and then examine the potential implications of cholinergic system dysfunction in patients with CKD.

## THE CHOLINERGIC SYSTEM AND COGNITIVE FUNCTION

### The physiology of the cholinergic system

The cholinergic pathways involve multiple modalities related to the classical neurotransmitter acetylcholine (ACh), including regulation of its production and degradation and its ability to modulate cellular pathways through interactions with specific receptors. Cells involved in cholinergic signalling can be divided into two categories: cholinergic cells, which produce ACh (e.g. cholinergic neurons [13]), and cholinoceptive cells, which possess receptors and can respond to available ACh (e.g. various cell types in the kidney [14]). Similarly, drug substances are classified as cholinergic or anti-cholinergic based on their agonistic or antagonistic effects on cholinergic receptors, which are divided into two types: ligand-gated ion channels, known as nicotinic acetylcholine receptors (nAChRs; subtypes α1–7, α9–10, β1–4, δ, ε, γ), and G-protein-coupled muscarinic acetylcholine receptors (mAChRs; subtypes M1–5) [15].

**Figure 1:**
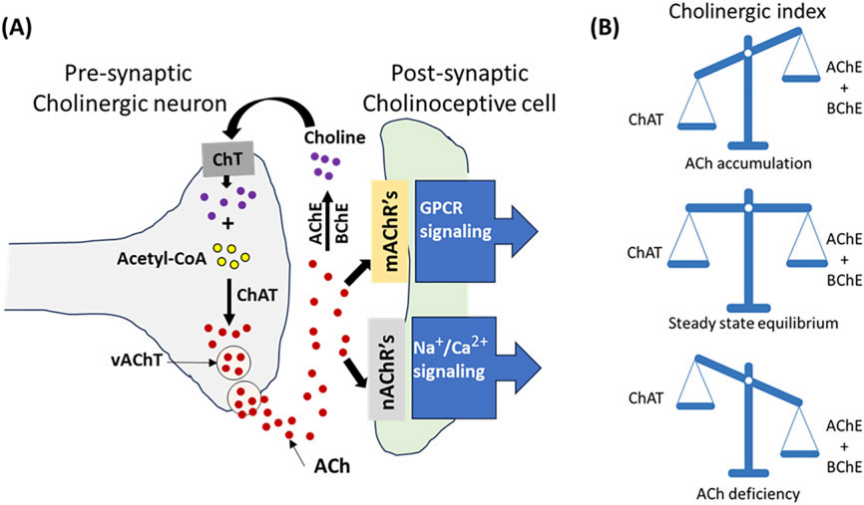
The cholinergic system and ACH equilibrium. **(A)** The production and clearance of ACh. ChAT catalyses ACh formation from choline and acetyl CoA in the cytosol of the presynaptic cholinergic neuron. The vAChT loads the ACh into synaptic vesicles that are then fused with the synaptic cleft cell membrane to release ACh into the synapse. Free ACh can either engage various receptors—nAChRs or mAChRs—present in the cholinoceptive cell or become degraded by AChE or BChE into choline and acetate. The available choline undergoes reuptake by the cholinergic neuron through the high-affinity ChT to continue the process of ACh production. In contrast, depending on their availability and distribution, nAChRs and mAChRs can initiate various signalling pathways in the cholinoceptive cells that may lead to various biological effects. **(B)** The cholinergic index, which represents the balance between ACh production and clearance. The steady-state equilibrium is maintained when the production and clearance rates of ACh are in tandem with each other, wherein ChAT activity represents ACh production, whereas ACh clearance is represented by the combined efforts of AChE and BChE. A change in ACh production or clearance may lead to situations of ACh accumulation or deficiency.

Under normal physiological conditions in the brain, most ACh is produced inside cholinergic neurons primarily by the enzyme choline acetyltransferase (ChAT) in the cytosol, and to a lesser extent by carnitine acetyltransferase (CRAT) within the mitochondria. Choline and acetyl co-enzyme A (CoA) are converted into ACh by ChAT, after which ACh is then released into synaptic vesicles by the vesicular acetylcholine transporter (vAChT). These vesicles release ACh into the synaptic cleft after fusing with the presynaptic membrane, where ACh interacts with nicotinic or muscarinic receptors on cholinoceptive cells to trigger downstream signalling. The type of ACh receptor present on the cholinoceptive cells determines the specific signalling pathway and biological outcome in different cell types [15]. Simultaneously, ACh in the synaptic cleft is broken down by two cholinesterases—acetylcholinesterase (AChE) and butyrylcholinesterase (BChE)—releasing choline. This choline is then reabsorbed by presynaptic cholinergic neurons through the high-affinity choline transporter (ChT), ensuring an adequate supply for ChAT activity and further ACh production (Fig. 1A). An imbalance in ACh production or degradation can alter ACh availability, which is reflected in the cholinergic index (the ChAT/ChE, representing the balance between ACh production and degradation) (Fig. 1B).

In the CNS, ACh is produced primarily by cholinergic neurons, whose cell bodies are clustered in the basal forebrain. However, their projections extend to various brain regions, including the cortex and hippocampus—two key areas involved in memory and cognition [13]. The critical role of ACh signalling cognitive function is well established and is particularly evident in dementia disorders, where ACh signalling is significantly reduced [16, 17]. In the peripheral nervous system, ACh plays a vital role in regulating various organs, mainly through the parasympathetic nervous system. Vagus nerve–mediated signalling has been extensively documented to modulate organ function, including the regulation of kidney activity [18–20].

### Association between the cholinergic system and cognition

ACh regulates neuronal excitability throughout the nervous system by acting on both the cys-loop ligand-gated nAChRs and mAChRs. The hippocampus, a key brain region for learning and memory, expresses both types of receptors. Activation of these receptors by cholinergic input from the basal forebrain regulates synaptic communication essential for cognitive function [21]. As early as the 1970s, it was shown that cognitive decline is associated with central cholinergic dysfunction and neurodegeneration of the basal forebrain [22–24]. Studies also showed that cholinergic agonists improve and antagonists impair memory [25]. AD, characterized by progressive memory loss and cognitive decline, has been strongly linked to central cholinergic dysfunction originating in the basal forebrain, from which cholinergic neurons project to several brain regions, including the cerebral cortex and hippocampus. ACh plays a crucial role in regulating cognitive functions associated with these regions [26]. This accumulated knowledge led to formulation of the cholinergic hypothesis, which proposes that cholinergic neuron degeneration in AD is directly associated with cognitive impairment [27]. Later studies confirmed this hypothesis, showing that the cholinergic neurons in the basal forebrain degenerate early in AD [28, 29] and that reduced basal forebrain volume correlates with cognitive decline [30, 31]. Based on the cholinergic hypothesis, cholinesterase inhibitors (ChEIs), which enhance ACh signalling, were developed in the 1990s as a treatment for mild to moderate AD [32, 33].

Several factors influence the cholinergic system, with one of the most critical being the neurotrophin nerve growth factor (NGF), which is essential for the survival and plasticity of central cholinergic neurons [34]. Under normal physiological conditions, NGF enhances cholinergic signalling and anti-inflammatory pathways and regulates amyloid precursor protein (APP) β-processing, thereby reducing the formation of pathological amyloid-β (Aβ) [35, 36]. However, NGF metabolism has been reported to be impaired in AD, making NGF a promising target for therapeutic intervention [37, 38]. In addition, altered NGF maturation, as observed in AD, may lead to inefficient axonal transport and disrupted signalling, which can compromise homeostatic control and contribute to cholinergic dysfunction [38, 39].

In summary, the central cholinergic system plays a pivotal role in cognition. Understanding its mechanisms is crucial for addressing neurodegenerative conditions and, as this review will explore, may also provide insights into conditions where the peripheral cholinergic system plays a dominant role.

## THE CHOLINERGIC SYSTEM IN KIDNEY DISEASE

### The impact of CKD on the cholinergic system and cognitive function

The association between cholinergic system damage and cognitive impairment in CKD is an area of growing interest in medical research [20, 40]. CKD is increasingly recognized as a risk factor for cognitive impairment and dementia. The cognitive decline associated with CKD affects various domains, including memory, attention, executive function and visuospatial abilities, significantly reducing patients’ quality of life [41]. A key contributor to cognitive dysfunction in CKD is damage to the cholinergic system. Several mechanisms in CKD contribute to cholinergic dysfunction, thereby exacerbating cognitive decline. Table 1A summarizes key findings from both animal and human studies. One primary factor is the accumulation of uraemic toxins due to impaired kidney function. These toxins can cross the blood–brain barrier, interfering with the cholinergic system by reducing the activity of cholinergic neurons in the basal ganglia [42], downregulating neuronal nAChRs [43] and impairing ACh synthesis and release. A recent animal study [44] demonstrated that CKD mouse models exhibit psychomotor behavioural abnormalities and blood–brain barrier disruption. This was accompanied by impaired acetylcholinesterase activity, reduced neuronal arborization in multiple brain regions and decreased dendritic spine density. Specific brain regions also showed evidence of oxidative stress, inflammation and mitochondrial dysfunction, contributing to neurochemical and structural changes [45]. Furthermore, three cross-sectional human studies [46–48] involving dialysis patients reported reduced brain choline concentrations as measured by magnetic resonance spectroscopy. But it is important to note that although cholinergic dysfunction is observed in both AD and CKD, the underlying pathways leading to this dysfunction remain largely unknown. Recent studies suggest that the *APOE4* genotype and Aβ peptides may play a role in disrupting cholinergic mechanisms in the AD brain [49, 50]. However, similar findings have not yet been reported in CKD. In contrast, CKD patients have been found to exhibit elevated levels of Aβ in both plasma and the brain [51], possibly indicating impaired peripheral clearance of amyloid peptides.

**Table 1:** Main studies investigating the cholinergic system's role in patients with kidney disease.

Study	Design	Population	Exposure	Outcome	Cholinergic system test	Results
**(A) Impact of kidney disease on the cholinergic system**						
Animal studies						
Wang *et al.*, 2023 [42]	Randomized animal experiment	Male Sprague–Dawley rats randomized into control, early-stage CKD and late-stage CKD groups (10 each)	CKD induced by radical nephrectomy of the kidney	Cognitive abilities; hippocampal expression of BDNF, ChAT and synaptophysin (SYP)	ChAT	The progression of CKD-induced cognitive impairment is associated with the downregulation of ChAT, BDNF and SYP expression
Mazumder *et al.*, 2019 [44]	Randomized animal experiment	Swiss Albino male mice randomized into CKD and control groups (42 each)	CKD induced by receiving adenine-rich diet	Motor behavioural test, cognitive behavioural test, and activities AChE	AChE	CKD mice show significant declines in motor and cognitive behavioural tests. AChE activity is visibly reduced in different brain regions, including the prefrontal cortex, cerebral cortex, striatum, amygdala, hippocampus and substantia nigra
Ballesta *et al.*, 2012 [43]	Animal experiment	Male Wistar rats with renal failure	CKD induced by nephrectomy (moderate and severe renal failure)	Cognitive functions, the levels and functionality of α7 nAChRs in the brain	nAChRs	Impairments in short-term memory but not in long-term memory. A significant reduction in nicotinic receptors in the brain has been observed, which correlated with the extent of renal impairment
Human studies						
Anazodo *et al.*, 2023[46]	Cross-sectional study	17 HD patients, mean age 63 ± 13 years, 58.8% male	Before and during the final 60 min of HD	MRI, DTI and MRS to measure brain structure and neurochemistry	Proton MRS to measure choline concentrations	Dialysis leads to changes in white matter alterations indicative of cytotoxic oedema, accompanied by an increase in global brain volumes. There is a decrease in brain metabolite concentrations, including NAA and choline
Zhang *et al.*, 2017 [47]	Cross-sectional study	Pre-dialysis (*n* = 13), HD (*n* = 13), PD (*n* = 12), ages 27–74 years	Different dialysis therapies (pre-dialysis, HD and PD)	Metabolite concentrations in the bilateral amygdala, hippocampus and unilateral ACC	Proton MRS (1H-MRS) to measure choline-containing compounds	Choline-containing compounds, myo-inositol and glutamate and glutamine levels in the ACC were elevated in HD patients compared with pre-dialysis and PD patients. The metabolite concentrations correlated with the psychological status, which was better in HD than in pre-dialytic and PD patients
Tryc *et al.*, 2011 [48]	Cross-sectional study	23 non-dialysed patients with CKD stages 4 and 5, 15 dialysed patients; 63 healthy controls	Pre-dialysis or HD	Cognitive function, cerebral metabolite concentrations	MRS to measure choline concentrations	MRS changes were mainly observed in white matter. Choline concentration and combined N-acetylaspartate and N-acetylaspartylglutamate concentration levels decreased only in dialyzed patients. Both patient groups exhibited memory, learning and attention issues, with dialyzed patients showing more severe attention deficits
Lepping *et al.*, 2021 [55]	Prospective, longitudinal, cohort study	22/29 patients ages 30–70 years underwent KT and were followed for 12 months post-KT. 19 age-matched healthy controls	KT	Neurochemical concentrations (N-acetylaspartate, choline, glutamate, glutamine, myo-inositol and total creatine)	MRS to measure N-acetylaspartate, choline, glutamate, glutamine, myo-inositol and total creatine	Neurochemicals choline and myo-inositol were elevated pre-KT but normalized after KT
Fiorina *et al.*, 2012 [52]	Prospective, longitudinal, cohort study	15 ESRD plus T1D patients, 23 patients with ESRD plus T1D after KT (*n* = 9) and KP (*n* = 14) and 8 age-matched controls	KT and KP compared with ESRD and T1D patients	Changes in cerebral morphology (assessed via MRI) and cerebral metabolism (assessed via 1H-MRS)	1H-MRS to measure choline-containing compounds	MRI revealed a higher incidence of cerebrovascular disease in ESRD plus T1D patients. Brain 1H-MRS indicated lower NAA:choline ratio in ESRD plus T1D, KT and KP patients. The NAA:creatine ratio was lower in ESRD plus T1D patients compared with KP and controls. In KP patients, most of these features appeared to normalize after 5 years of sustained normoglycaemia
Sasaki *et al.*, 2006 [53]	Prospective, longitudinal, cohort study	19 patients with CKD and 21 age-matched healthy controls	The initiation of HD	Cerebral metabolite concentrations	Proton MRS to measure choline concentrations	Patients with CKD showed elevated choline:creatine ratios in both grey and white matter. After 18 months of HD, the choline:creatine ratio decreased in the grey matter and tended to decline in the white matter. Choline regulates cerebral metabolism to address serum osmotic changes in CKD, and HD normalize this metabolism
**(B) Impact of the cholinergic system on kidney disease**						
Animal studies						
Uni *et al.*, 2020 [12]	Animal experiment	Mice models of AKI	VNS device; splenectomy; selective α7nAChR agonist GTS-21	Renal function, renal histology, renal injury marker (Kim-1) and the expression of cytokines and chemokines	VNS device, AChR agonist	VNS reduced cytokine and chemokine expression and lessened AKI. This effect was eliminated by splenectomy and restored with the adoptive transfer of GTS-21-treated macrophages
Inoue *et al.*, 2019 [61]	Animal experiment	Mice model of IRI-AKI	Ultrasound or VNS	Renal function, renal histology, acute tubular necrosis score, cytokines and gene expression	Ultrasound or VNS	Ultrasound or VNS activated α7nAChR-positive peritoneal macrophages and reduced kidney injury in mice from IRI. Ultrasound or VNS increased the expression of Hes1, a DNA-binding protein, in peritoneal macrophages
Inoue *et al.*, 2016 [60]	Animal experiment	Mice model of IRI-AKI	VNS device 24 h before IRI	Renal function, renal histology, renal injury marker (Kim-1) and haematoxylin and eosin staining of kidney tissues and cytokines	VNS device	VNS 24 h before IRI attenuated AKI and decreased plasma TNF. VNS-mediated attenuation of AKI and systemic inflammation depends on α7nAChR-positive splenocytes
Sadis *et al.*, 2007 [62]	Randomized animal experiment	Mice model of AKI	Nicotine administration	Renal function, renal tubular damage and inflammatory response	AChR agonist	Nicotine reduced renal tubular damage and inflammation, while also decreasing tubular cell apoptosis and the proliferative response in pretreated mice
McGiff *et al.*, 1967 [66]	Animal experiment	Male mongrel dogs	ACh	Renal blood flow	ACh in different doses	ACh increased renal blood flow in the canine kidney by releasing norepinephrine during sympathetic nerve activity
Maloy *et al.*, 2016 [67]	Randomized animal experiment	SLE mice (NZBWF1)	CNI-1493 administered intraperitoneally to stimulate the vagus nerve	Mean arterial pressure, renal blood flow, renal vascular resistance and albuminuria	VNS	CNI-1493 treatment reduced mean arterial pressure, decreased albumin excretion, increased renal blood flow and lowered renal vascular resistance in SLE mice
Wu *et al.*, 2021 [68]	Animal experiment	Male Sprague–Dawley rats	Selective α7nAChR agonist GTS-21, v agotomy	Blood pressure, baroreflex sensitivity, NF-κB activation and fibrosis in NRK-52E cells	AChR agonist	GTS-21 reduced AngII-induced hypertension, improved baroreflex sensitivity and decreased fibrosis and inflammation. *In vitro*, GTS-21 suppressed NF-κB activation and reduced AngII-induced epithelial–mesenchymal transition, inflammation and fibrosis
Human studies						
Xu *et al.*, 2023 [70]	Prospective, longitudinal, cohort study	Alzheimer's disease patients (*n* = 11 898, mean age 80 years, 64% women, mean eGFR 68 ml/min/1.73 m^2^)	ChEI	CKD progression	ChEIs	ChEI use was linked to a reduced risk of CKD progression
Hilderman, *et al.*, 2020 [69]	Pilot study	HD patients (7 males, 5 females, age range 47–86 years)	Treatment with VNS device for 4 weeks	CRP, white blood cells, TNF, IL-1 and IL-10	VNS device	Cytokine changes did not reach statistical significance

ACC: anterior cingulate cortex; AKI: acute kidney injury; BDNF: brain-derived neurotrophic factor; DTI: diffusion tensor imaging; ESRD: end-stage renal disease; HD: haemodialysis; IL; interleukin; IRI, ischaemia–reperfusion injury; KP: kidney–pancreas transplantation; KT: kidney transplant; MRI: magnetic resonance imaging; MRS: magnetic resonance spectroscopy; PD: peritoneal dialysis; SYP: synaptophysin; TNF: tumour necrosis factor; T1D: type 1 diabetes.

CKD is characterized by chronic systemic inflammation, which may extend to the CNS. Neuroinflammation has been implicated in the pathogenesis of cognitive decline in CKD. The cholinergic anti-inflammatory pathway, mediated by the vagus nerve, plays a key role in regulating inflammation in both the central and peripheral nervous systems. Dysfunction of this pathway in CKD can result in excessive neuroinflammation, further exacerbating cognitive impairment [40]. CKD is also associated with vascular diseases, including small vessel disease and cerebral microbleeds. Vascular injury disrupts the integrity of the blood–brain barrier and impairs cerebral perfusion, leading to cerebral hypoperfusion and ischaemia. CKD-related factors, such as metabolic disorders, oxidative stress and uraemic toxicity, can induce structural and functional changes in the brain. These changes, coupled with cholinergic system dysfunction, may impair neuronal plasticity and synaptic transmission, contributing to the cognitive deficits observed in CKD.

One cohort study using magnetic resonance imaging (MRI) demonstrated a higher incidence of cerebrovascular disease in patients with kidney failure compared with healthy individuals. Magnetic resonance spectroscopy (MRS) further revealed a lower N-acetylaspartate (NAA):choline ratio in patients with kidney transplants, indicating metabolic disturbances in the brain [52]. Another study involving non-dialysis patients with chronic renal failure found an elevated choline:creatine ratio in both grey and white matter compared with healthy controls. Interestingly, after ≈18 months of haemodialysis (HD), the elevated choline:creatine ratio in the grey matter significantly decreased, with a similar trend observed in the white matter [53]. The cholinergic system also plays a crucial role in regulating cerebral blood flow. Dysfunction of this system may worsen cerebral hypoperfusion, further contributing to cognitive decline in CKD. Notably, interventions such as HD or kidney transplants may help normalize brain metabolism [54, 55]. These mechanisms highlight the impact of CKD on brain function and may contribute to cognitive decline.

## THE IMPACT OF THE CHOLINERGIC SYSTEM ON RENAL FUNCTION

Recent studies indicate parasympathetic innervation of renal tissues, suggesting direct cholinergic involvement in kidney function [56]. Furthermore, ACh production has been reported in mouse podocytes [57] and rabbit cortical cells [58], although confirmation in human cells or organs is still pending. Emerging evidence also suggests that the cholinergic system plays a role in the pathophysiology of both AKI and CKD. Table 1B summarizes key findings from animal and human studies on this topic.

One key mechanism is the cholinergic anti-inflammatory pathway, mediated by the vagus nerve, which has been shown to exert anti-inflammatory effects throughout the body, including the kidneys. Cholinergic neurons in the forebrain, which are known to regulate peripheral inflammation, have been shown to be affected in AD, potentially contributing to increased systemic inflammation [59]. Animal studies have demonstrated that vagus nerve stimulation can reduce AKI by activating cholinergic anti-inflammatory pathways via α7 nACHRs (α7nAChRs) on splenic macrophages [12, 60] and α7nAChR-expressing peritoneal macrophages [61]. Another study [62] demonstrated that treatment with cholinergic agonists significantly reduced tumour necrosis factor α protein expression and renal leucocyte infiltration in the context of ischaemia–reperfusion injury. Additionally, cell culture studies have identified functional nAChRs in glomeruli [63], inner medullary collecting duct cells [64] and proximal tubule epithelial cells [11]. One study demonstrated that interfering with the M3 mAChR—one of the five G-protein-coupled receptor subtypes (M1–M5) responsible for ACh's physiological functions—improved renal fibrosis in transforming growth factor β–treated NRK-49F cells [11]. Moreover, ACh plays a key role in regulating renal haemodynamics and sodium excretion [65]. Changes in ACh activity, such as the use of anticholinergic drugs, may affect kidney function. Dysregulation of the cholinergic system is thought to contribute to the renal dysfunction observed in CKD. For example, studies have shown that injecting different doses of ACh into the renal arteries increases renal blood flow in dogs [66]. Additionally, vagus nerve stimulation has been shown to protect the kidneys and prevent the development of hypertension in mice with chronic inflammation [67]. The cholinergic system also interacts with other physiological systems, such as the renin–angiotensin–aldosterone system and the sympathetic nervous system. Imbalances in these systems have been implicated in CKD progression and the development of renal complications. Cholinergic system dysfunction may disrupt the regulation of these pathways, further influencing renal function and disease progression. For instance, angiotensin II (AngII) promotes renal fibrosis, while the vagus nerve–mediated cholinergic anti-inflammatory pathway helps mitigate AngII-induced hypertension by inhibiting nuclear factor κB (NF-κB) activation, thereby reducing fibrosis and inflammatory responses [68]. This pathway inhibits NF-κB activation, thereby reducing renal fibrosis and inflammatory responses. Given the intricate interplay between the cholinergic system and renal function, targeting this system may offer a novel therapeutic approach for CKD patients. Two clinical studies have investigated this potential. In one pilot study [69], a vagus nerve stimulation (VNS) device was used in HD patients, but no statistically significant changes in cytokine levels were observed after 4 weeks. In contrast, another cohort study examined the use of ChEIs and found that these drugs were significantly associated with slower progression of CKD [70]. This suggests a possible mechanism by which ChEIs may help manage CKD. Further research is needed to clarify the precise role of cholinergic dysfunction in CKD and to identify potential therapeutic targets for intervention.

## CLINICAL APPLICATIONS AND FUTURE RESEARCH DIRECTIONS

Drugs that directly modulate the cholinergic system can induce undesirable effects, including adverse reactions affecting the CNS. Medications with anticholinergic effects are commonly prescribed to manage various conditions, such as overactive bladder, nausea and vomiting, excessive gastric acid production, intestinal motility disorders, psychosis, depression, muscle spasms, allergies and chronic obstructive pulmonary disease [71]. However, numerous reports have linked the use of anticholinergic drugs to adverse outcomes, such as hospitalization, cognitive impairment and increased mortality [72–74]. For example, a study involving >19 000 participants ≥70 years of age, all initially free from dementia, demonstrated that a higher anticholinergic burden predicted a decline in cognitive function over time [75]. In a large cohort of CKD patients with an estimated glomerular filtration rate (eGFR) <60 ml/min/1.73 m^2^, Mouheb *et al.* [76] reported that 52% of patients were prescribed at least one drug with anticholinergic properties. Furthermore, patients with a high anticholinergic burden had a significantly higher likelihood of cognitive impairment compared with those without an anticholinergic burden, even after adjusting for sociodemographic factors, comorbidities, laboratory data and the number of daily medications taken. Given that CKD patients are particularly prone to polypharmacy, it is crucial to carefully re-evaluate their prescriptions to identify and, where possible, reduce the use of medications with anticholinergic properties.

**Figure 2:**
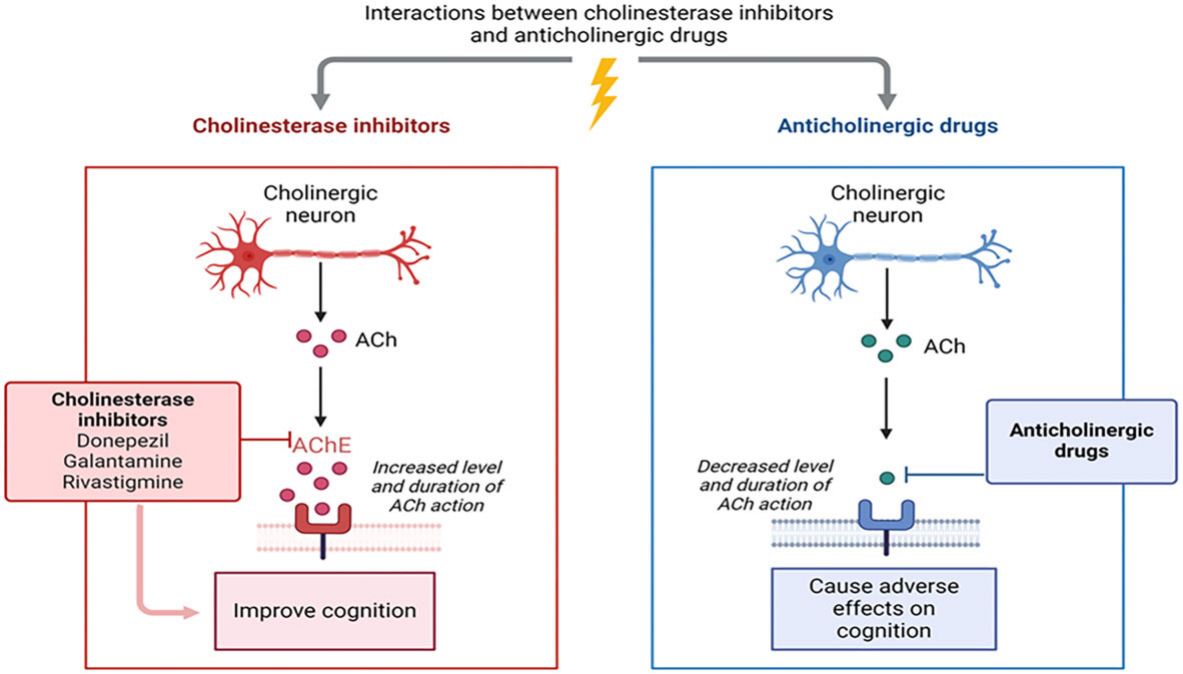
Mechanism of action of cholinergic and anticholinergic drugs.

In contrast, therapeutic options that stimulate the cholinergic system may benefit both cognitive and renal function in CKD patients. ChEIs such as donepezil, galantamine, and rivastigmine remain the primary pharmacological treatment for AD to address symptoms related to memory, thinking, language, judgment and other cognitive functions [41] (Fig. 2). Intriguingly, there is growing evidence linking ChEI use to cardio- and cerebrovascular protection. A recent meta-analysis of nine cohort studies reported a 37% reduction in cardiovascular events and cardiovascular mortality associated with ChEI use [77]. Moreover, our research has shown that ChEI treatment is linked to significant reductions in mortality and a lower risk of myocardial infarction [78], stroke [79] and renal dysfunction [70]. These protective effects might be attributable to the anti-inflammatory properties of ChEIs and suggest that their benefits extend beyond the cholinergic system in the brain [80].

To the best of our knowledge, the impact of ChEIs on cognition in CKD patients has not yet been evaluated. However, future clinical trials could investigate the potential beneficial effects of cholinergic system stimulation—whether through pharmacological treatment or VNS devices—on both cognitive and renal function in CKD patients.

## CONCLUSION

Understanding the connection between kidney dysfunction and cognitive impairment could deepen our understanding of various cognitive disorders. The brain's cholinergic system plays a potentially central role in ongoing research on normal cognition and age-related cognitive decline, including dementias such as AD, as well as in the regulation of peripheral inflammation. This system should be carefully considered in individuals with CKD. In addition, the cholinergic system may contribute to the pathophysiology of CKD, while altered kidney function in CKD can, in turn, affect brain functions through various mechanisms discussed in this review. In both AD and CKD, sustaining cholinergic pathways may improve cognitive outcomes by acting directly on both central and peripheral systems and by mitigating the effects of CKD-related dysfunction.

## Data Availability

Not applicable.

## APPENDIX

The CONNECT collaborators: Giovambattista Capasso, Alexandre Andrade, Mustafa Arici, Maie Bachmann, Matthew Bailey, Michelangela Barbieri, Mickaël Bobot, Annette Bruchfeld, Inga Arune-Bumblyte, Daiva Rastenytė, Antonello Calcutta, Giovanna Capolongo, Sol Carriazo, Michele Ceccarelli, Adrian Constantin Covic, Ananya De, Pilar Delgado, Nicole Endlich, Matthias Endres, Fabrizio Esposito, Michele Farisco, Quentin Faucher, Ana Carina Ferreira, Andreja Figurek, Denis Fouque, Casper Franssen, Ivo Fridolin, Sebastian Frische, Liliana Garneata, Loreto Gesualdo, Konstantinos Giannakou, Olivier Godefroy, Aleksandra Golenia, Dimitrios Goumenos, Eugenio Gutiérrez Jiménez, Gaye Hafez, Ewout Hoorn, Pedro Henrique Imenez Silva, Raafiah Izhar, Dearbhla Kelly, Shelli Kesler, Aleksandra Klimkowicz-Mrowiec, Samuel Knauss, Justina Kurganaite, Hélène Levassort, Sophie Liabeuf, Jolanta Malyszko, Laila-Yasmin Mani, Gianvito Martino, Ziad Massy, Christopher Mayer, Armida Mucci, Alma Mutevelic-Turkovic, Rikke Nielsen, Dorothea Nitsch, Alberto Ortiz, Vasileios Panagiotopoulos, Despoina Karasavvidou, Giuseppe Paolisso, Bojana Pejušković, Marion Pepin, Alessandra Perna, Andrea Perrottelli, Vesna Pešić, Pasquale Pezzella, Merita Rroji (Molla), Ivan Rychlík, Giorgos Sakkas, Mariadelina Simeoni, Maria José Soler Romeo, Goce Spasovski, Ana Starčević, Gioacchino Tedeschi, Francesco Trevisani, Robert Unwin, Evgueniy Vazelov, Carsten Alexander Wagner, Franca Wagner, Christoph Wanner, Andrzej Wiecek, Hong Xu, Miriam Zacchia, Lefteris Zacharia, Irene Zecchino, Carmine Zoccali, Francesco Mattace-Raso, Karl-Hans Endlich, Norberto Perico, Giuseppe Remuzzi, Francesco Trepiccione, Mark Okusa, Vincenzo Di Marzo, Peter Blankestijn, Kai-Uwe Eckardt, Maximilian Konig, Ron Gansevoort, Hassan Askari, Brian Hansen, Sunna Snaedal, Elena Cuiban, Edoardo Caporusso, Vincenzina Lo Re, Jonathan Roiser, Kerry Rosenberg, Alvino Bisecco, Laura Denby, Onkar Prakash Kulkarni, Kumar Sharma, Subrata Debnath, Afaf Jaafar, Anna Capasso, Michele Mulholland, Biruh Workeneh, Anna Iervolino, Simon Fraser, Isabelle Frey-Wagner, Annachiara Pastore, Romaldas Mačiulaitis, Antonio De Donato and Ana Farinha.

## Notes

CONNECT Action (see Appendix)
